# Feasibility of using infant testing during immunization to estimate HIV mother-to-child-transmission rates in Zambia

**DOI:** 10.1186/s12879-021-06892-0

**Published:** 2021-12-09

**Authors:** Joseph Simbaya, Patricia Funjika, Arthur Moonga, John Mwale, Chipepo Kankasa

**Affiliations:** 1 Institute of Economic and Social Research (University of Zambia), Munali Road, 10101 Lusaka, Zambia; 2 Institute of Development and Research, Mosi-oa-Tunya Road, 10101 Lusaka, Zambia; 3 Zambia National HIV/AIDS/TB Council, Independence Avenue, 10101 Lusaka, Zambia; 4 University Teaching Hospital HIV AIDS Program (UTH-HAP), Burma Road, 10101 Lusaka, Zambia

**Keywords:** HIV, Mother-to-child transmission, Africa, Children

## Abstract

**Background:**

This study piloted the feasibility of infant testing in immunization services as a strategy for estimating MTCT rates among the population of HIV exposed infants at national and subnational levels in Zambia.

**Methods:**

The study recruited a cross-sectional nationally representative sample of 8042 caregiver-baby pairs in 38 high volume immunization sites in 7 towns across 3 provinces of Zambia. All mothers who brought their children below the age of one year for immunization at the study facilities were invited to participate in the study. All consenting mothers were interviewed and blood drawn from their babies for; rapid HIV antibody test to determine exposure and DNA PCR test for samples of all HIV-exposed babies to determine HIV infection.

**Results:**

Of 8042 recruited caregiver–baby pairs, 1409 (17.5%) babies were HIV-exposed. Approximately 90.2% of all mothers of HIV exposed infants reported that they attended ANC visits more than two times and facility based deliveries stood at 91.6%. Exclusive breastfeeding among HIV exposed infants reduced with increase in age of infant; it was highest at 6 weeks (82.2%) followed by 10 weeks (74.0%) and 14 weeks (58.2%). MTCT rates were relatively lower than what was reported before in subnational studies and stood at 4.7% among Penta 1 seekers, 2.8% among Penta 2 seekers, 2.1% among Penta 3 seekers and 5.0% among Measles vaccination seekers. The overall MTCT rate stood at 3.8%. About 48.1% of HIV positive babies were male compared to 51.9% females. Babies of mothers below the age of 25 years accounted for almost half (51.9%) of all HIV infected babies in the study. Reported exclusive breastfeeding among HIV positive babies was 77.8% for Penta 1 seekers, 75.0% for Penta 2 seekers and 100% for Penta 3 seekers.

**Conclusions:**

The study succeeded in estimating the MTCT rates using infant testing in immunization services, thereby demonstrating that it is feasible to use routine infant testing in immunization services as a strategy for estimating MTCT rates among the population of HIV-exposed infants in countries with high HIV burden and immunization coverage.

## Background

Vertical transmission of Human Immunodeficiency Virus (HIV) is the most common source of paediatric HIV infection in Zambia [1]. In 2016, around 8900 children became newly infected with HIV in Zambia indicating a 56% decline from 20,000 new paediatric infections in 2009 [2, 3]. Mother-to-Child Transmission of HIV (MTCT) ranges from 15 to 45% in the absence of any intervention. MTCT can however be reduced to below 5% with effective interventions during pregnancy, delivery and breastfeeding. Intervention include inter alia; Antiretroviral Treatment (ART) for the HIV positive pregnant woman or mother and a short course of antiretroviral drugs for the exposed infant, facility based delivery, and appropriate breastfeeding practice [4, 5].

Zambia first introduced its rigorous Prevention of Mother-to-Child Transmission of HIV (PMTCT) programme in 1999 and has been committed to virtual elimination of MTCT since 2010 [6]. Zambia’s prevention and treatment of HIV infection guidelines requires the testing of all women receiving Antenatal Care (ANC) and enrolment of all those that are found positive into HIV care and treatment [7].

Despite Zambia’s continued efforts to eliminate MTCT, measuring of MTCT at national level remains a challenge despite the routine documentation of infant testing data through the PMTCT programme. Previous studies on efficacy of routine HIV testing data from PMTCT programmes have indicated that the data is largely unavailable and when it is available, it is usually incomplete, inaccurate and not available on time [8–12], thus partly explaining the reluctance to use it for estimating MTCT rates. Previous efforts to measure MTCT rates have therefore primarily focused on subnational levels.

Measurement of MTCT is critical in planning and meeting the needs of paediatric HIV prevention, care, and treatment services for children, as well as evaluating the effectiveness of PMTCT interventions [13, 14]. The Government of the Republic of Zambia and the international community are cognizant of the challenge in measuring MTCT. This study was therefore conducted with the aim of assessing the feasibility of infant testing in immunization services as a strategy for estimating MTCT rates among the population of HIV exposed infants (HEI).

The Zambia Programme on Immunization seeks to immunize infants against:

- Tuberculosis with the Bacille Calmette Guerin (BCG) vaccine at birth;
- Polio with oral polio vaccine (OPV) at 0 (birth), 6, 10 and 14 weeks and inactive polio vaccine (IPV) at 14 weeks;
- Diphtheria, Pertussis, Tetanus, Haemophilus Influenza type B, Hepatitis B with three doses of the Penta vaccine at 6, 10 and 14 weeks of age (DPTHibHep1, DPTHibHep2 and DPTHibHep3 respectively);
- Diarrhoea with Rota vaccine at 6 and 10 weeks;
- Streptococcal Pneumonia with the pneumococcal conjugate vaccine (PCV) at 6, 10 and 14 weeks
- Measles and Rubella vaccines at 9 and 18 months of age;

HIV testing for HEI in Zambia is aligned to immunization and is done at birth ( during BCG and OPV), 6 weeks (during OPV, DPT1 and Rota), 14 weeks (during OPV, IPV, and PCV), 9 months (during Measles and Rubella), 12 months, 18 months (during measles and rubella), and 24 months [7]. This study was guided by immunization service statistics which suggest that client flow is fairly evenly divided among the three Penta vaccines and Measles vaccine, with the highest proportion at DPTHibHep1 and the lowest at measles.

## Methods

This was a variant of sentinel surveillance study which followed a cross-sectional approach. The study took advantage of Zambia’s highly successful childhood immunization program which starts at birth (BCG) through to 18 months (measles and rubella). Using these services, the study recruited mothers and infants for HIV testing to provide data for measuring the MTCT rates. The study sought to obtain blood samples from 8000 infants under the age of 12 months distributed across 38 health facilities from across three provinces (Copperbelt, Lusaka and Southern provinces of Zambia). It was estimated that this would yield a sample of approximately 1240 HEI.

The sample of 1240 was determined using an average HIV prevalence among women 15–49 of 21.6% in 2007 [15] and adjusting for lower prevalence among pregnant women than non-pregnant women (11.6% vs. 16.6% nationally) and the pregnancy rate (8% currently pregnant), it was estimated that 15.5% of women with a recent birth are HIV-positive. Assuming no differential mortality, equal likelihood of seeking vaccination and equal refusal rates, 1,240 (15.5%) out of 8000 infants tested would be HIV-exposed. The precision of the estimated age-specific MTCT rates would depend on the age distribution of HIV-exposed infants in the sample.

In light of national vaccination norms promoting Penta vaccine booster shots (Penta-2 and Penta-3) at 4-week intervals, data collection at any one vaccination site was limited to a 4-week period to avoid re-peat testing of infants. Participants were concurrently recruited at all vaccination sites in the study until a total sample of 8000 consenting participants was achieved.

During the data collection period, all consenting caregivers of infants between 0 and 12 months of age who were receiving vaccinations in study sites were recruited. Inclusion criteria for care-givers were: Caregiver of age 16 and over, attending vaccination for DPT1, DPT2, DPT3 or measles with infants younger than 12 months of age; and physically and mentally capable of providing informed consent for the interview.

This study involved interviewing the caregivers and taking blood samples from participating infants and testing them for both HIV antibodies (all recruited infants) and HIV DNA (infants with positive antibody tests). As part of obtaining informed consent, all caregivers were informed about the need for and difference between the two tests and that the presence of HIV antibodies in the baby’s blood meant that the mother was HIV-positive. However, anti-body test results were only availed to the caregivers on request and upon receiving adequate counselling. For quality assurance and infant safety purposes, the study used certified HIV counsellors and Nurses working at the respective health facility with experience in DBS collection. All study counsellors and nurses received additional training from the research team to ensure that they had the knowledge and skills to educate mothers about the testing procedures and to properly take and preserve infant blood samples. To avoid irritating the child, only one sample was drawn from which an antibody test was done to determine exposure as well as DBS to determine actual HIV infection.

One ethical consideration when testing babies for HIV exposure and infection is psychological distress that could be experienced by mothers when they learn that they and/or their infants are HIV-positive. The study team worked with the facility staff to ensure that certified facility counselors were in place to provide pre and post-test counseling to study participants, and to provide referrals for immediate prophylaxis or treatment to mother-baby pairs with positive HIV test results. Mother-baby pairs with positive HIV results were therefore recruited into treatment and care and the mother provided with continuous adherence assessment and counselling to ensure that treatment was taken as prescribed. Another ethical consideration for this study was that the study was conducted in a health facility where power dynamics are likely to be evident between health workers and mothers of infants. To ensure that participation in the study was voluntary, mothers were introduced to the study after the infant had received the vaccination and all healthcare services for the day. The Nurse referred the mother to the trained Research Assistant who independently sought consent from the mother. All consenting mothers were then interviewed and additional consent sought for drawing a blood sample from the infant, after which, all consenting mothers were referred to the laboratory for drawing the blood sample.

Data from the survey was entered using EpiData V3.1. Rapid test results to determine infant exposure were collected separately and linked to both the survey and the DBS data. The three datasets were merged for analysis. Frequency of key demographic and other health-seeking behaviour variables were performed. The MTCT rate was calculated as a proportion of infant blood samples with a positive PCR test result among infants with a positive HIV antibody test result; the numerator was the number of all PCR positive babies and the denominator was the number of all HIV exposed infants. The Pearson Chi-Square test was used to measure the association between HIV exposed infants and primary caregiver characteristics. Because of the small sample, the Fisher’s exact test was used for the HIV infected infants and demographic and health seeking characteristics of the primary caregivers. Reported P-values should be interpreted as descriptive rather than inferential statistics applicable to the larger population. A multivariate logistic model using robust standard errors was applied to assess the associations between infant HIV exposure and vertical transmission rates, and the population characteristics. We estimated odds ratios along with 95% confidence intervals separately for infant HIV exposure and infant HIV infection. Missing data were ignored. Analyses were performed using STATA version 16.

## Results

### Study population

The population for the study encompassed all caregiver–infant pairs attending immunisation for Penta vaccine 1, to 3, and measles vaccine for 9 months at the study sites. Of the 8522 caregiver–infant pairs that were approached, 8289 pairs consented for interviews and 8042 consented for infant HIV testing representing a 97.3% and 94.4% response rates for interviews and infant testing respectively as shown in Table 1. Copperbelt province had the highest interview response rate (98.6%) followed by Southern (97.0%) and Lusaka (96.0%) provinces and there was an almost equal distribution of infant testing response across provinces.

Table 1 further shows that Southern province recruited the highest number of participants (2960) followed by Copperbelt (2713) and Lusaka (2369). A total of seven districts participated in the study; Ndola, Kitwe and Chingola from Copperbelt Province, Livingstone, Choma, and Mazabuka from Southern Province, and Lusaka from Lusaka Province.

**Table 1:** Study participation rates per Province

Province	District	Clients	Contacted consenting				Refusals			
			For interviews		For blood test		Interview		For blood test	
			n	%	n	%	n	%	n	%
Copperbelt	Ndola	980	965	98.5	950	96.9	15	1.5	30	3.1
	Kitwe	987	970	98.3	950	96.3	17	1.7	37	3.8
	Chingola	939	930	99.0	813	86.6	9	1.0	126	13.4
	Total	2906	2865	98.6	2713	93.4	41	1.4	193	6.6
Southern	Livingstone	958	940	98.1	939	98.0	18	1.9	19	2.0
	Choma	1053	1033	98.1	1027	97.5	20	1.9	26	2.5
	Mazabuka	1105	1051	95.1	994	90.0	54	4.9	111	10.1
	Total	3116	3024	97.1	2960	95.0	92	3.0	156	5.0
Lusaka	Lusaka	2500	2400	96.0	2369	94.8	100	4.0	131	5.2
All		8522	8289	97.3	8042	94.4	233	2.7	480	5.6

### Social demographics

**Fig. 1:**
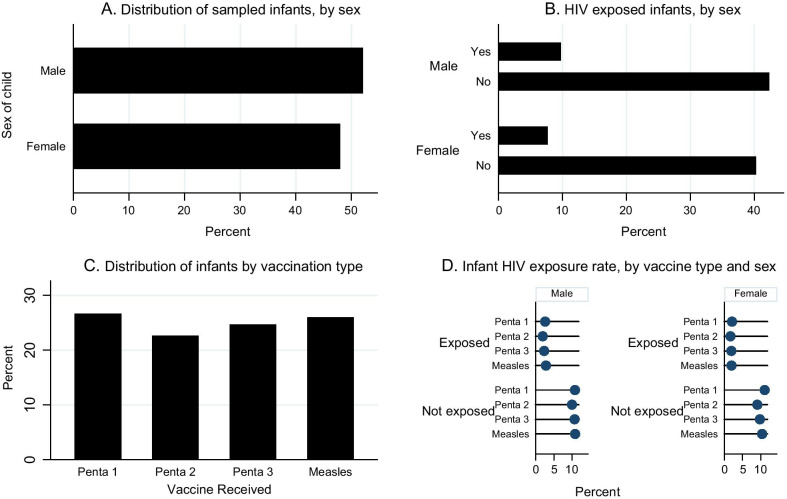
Infant Sex, HIV Exposure and Vaccination Type. **A** and **B** of the figure shows the distribution, in percentage, of sampled infants and HIV exposure by sex. **C** shows the infant distribution by vaccination type while **D** shows the infant HIV exposure rate disaggregated by vaccine type and sex

Male babies accounted for about 52.1% of the sample while female babies accounted for 47.9% (Fig. 1). Overall, slightly more babies recruited for the study came to the facility to receive Penta 1 (26.7%). The rest came for Penta 2 (22.6%), Penta 3 (24.7%) and Measles (26.0%).

The majority (47.1%) of the mothers were aged less than 25 years old. Similarly, the majority were married (81.6%), had secondary education (62.1%) and were not employed (67.2%) (Table 2).

**Table 2:** Infant HIV exposure by primary caregiver demographic characteristics

	N	%	Infants exposed	% exposed	P-value
Age of mother					
Less than 25	3,787	47.1	407	10.8	P < 0.001
25–29	1962	24.4	365	18.6	
30 +	2293	28.5	637	27.8	
Marital status					
Single	1303	16.2	194	14.9	P < 0.001
Cohabiting	55	0.7	14	25.5	
Married	6561	81.6	1,158	17.7	
Divorced	39	0.5	11	28.2	
Separated	57	0.7	17	29.8	
Widowed	27	0.3	15	55.6	
Highest level of education					
Primary	2300	28.6	476	20.7	P < 0.001
Secondary	4991	62.1	793	15.9	
Higher	457	5.7	85	18.6	
None	294	3.7	55	18.7	
Occupation (employment status)					
Formal employment	625	7.8	127	20.3	P < 0.001
Self-employed/business	1899	23.6	403	21.2	
Not employed	5405	67.2	865	16.0	
School	70	0.9	2	2.9	
Other	43	0.5	12	27.9	
Total	8042	100	1409	17.5	

### HIV infant exposure

The HEI accounted for 17.5% of the overall infants tested for HIV antibody. Slightly more male infants (18.8%) were HIV exposed than females (16.1%). Of all HEI (N = 1409), more male babies (55.9%) were exposed than female babies (44.1%). Slightly below half (45.2%) of all HEI had mothers above 30 years old while about a quarter (25.9%) had mothers in the age range of 25 to 29 years (Table 2).

**Fig. 2:**
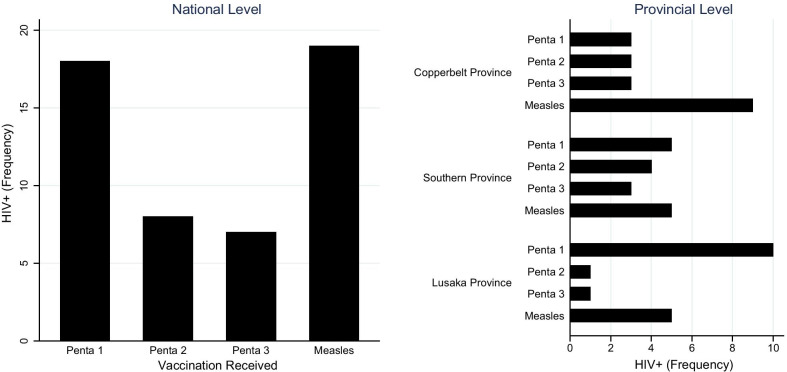
Number of HIV positive babies by district and infant age/immunization type. The figure shows the number of HIV positive babies by immunization type tested using DNA PCR. The distribution is illustrated at both national level and disaggregated by province

There were more HEI who came for Penta 1 (27.5%) and Measles (27.5%) vaccinations than those who came for Penta 2 (20.7%) or Penta 3 (24.3%). Overall, about 91.6% of all exposed infants were delivered in a health facility. Almost all mothers of exposed babies had tested for HIV (99%) and had attended three or more ANC visits (90.7%) (Table 4). Figure 2 presents the number of HII at national and provincial levels by immunization stage/type the baby came to receive at the time of drawing the sample. A total of 19 HIV positive results were detected in infants who came for Penta 1. Penta 2 and Penta 3 each had 9 babies testing positive to PCR test while Measles had 20.

### MTCT rates in immunization services

Of the 1409 exposed infants, 1389 samples were successfully tested for HIV using DNA PCR. A total of 12 samples were rejected and 4 had missing results at the time of study completion. A total of 52 samples were reactive to the PCR test representing an overall rate of transmission of 3.75% for infants aged 6 weeks to 9 months. The MTCT rates at 6 weeks, 10 weeks, 14 weeks and 9 months were 4.7%, 2.8%, 2.1% and 5.0% respectively (Table 3).

**Table 3:** Overall MTCT rates by Province and age of infant/immunization type

	Immunization type	Penta 1	Penta 2	Penta 3	Measles	Total
	Infant age	6 Weeks	10 Weeks	14 Weeks	9 months	
Province	Copperbelt Province	2.6	2.9	2.6	6.7	3.9
	Southern Province	4.1	4.4	2.4	2.9	3.4
	Lusaka Province	6.9	1.1	1.1	6.6	4.2
	All	4.7	2.8	2.1	5.0	3.8

Overall MTCT rates calculated as the proportion of PCR positive infants from the number of HIV exposed infants

The highest proportion of HII was among infants who came to seek Measles vaccination (36.5%) at 9 months followed by those who came to seek Penta 1 (34.6%) at 6 weeks. Penta 2 Penta 3 accounted for 15.4% and 13.5% of HII respectively. There were more female HII (51.9%) than male HII (48%). Slightly over half (51.9%) of all HII were among infants whose mothers were less than 25 years old. The majority (80.8%) HII were delivered in health facilities while 17.3% were delivered at home. The overall number of ANC attendance visits among mothers of HII was high (90.4% had at least 3 ANC visits) and so was reported exclusive breastfeeding (51.9%). Breastfeeding was measured as a percentage of infants 0–5 months of age who were fed exclusively with breast milk during the previous day [16].

**Table 4:** Characteristics of HEI and HII

	HEI	HII	MTCT	P-value*
Sex				
Male	788 (55.9%)	25 (48.1%)	3.2	P = 0.258
Female	621 (44.1%)	27 (51.9%)	4.4	
All	1,409 (100%)	52 (100%)	3.8	
Age of mother				
Less than 25	407 (28.9%)	27 (51.9%)	6.7	P = 0.002
25–29	365 (25.9%)	9 (17.3%)	2.5	
30 +	637 (45.2%)	16 (30.8%)	2.6	
All	1409 (100%)	52 (100%)	3.8	
Current vaccination				
Penta 1	387 (27.5%)	18 (34.6%)	4.7	P=0.115
Penta 2	292 (20.7%)	8 (15.4%)	2.8	
Penta 3	342 (24.3%)	7 (13.5%)	2.1	
Measles	388 (27.5%)	19 (36.5%)	5.0	
All	1409 (100%)	52 (100%)	3.8	
Place of delivery				
Home	113 (8.0%)	9 (17.3%)	8.1	P=0.010
Health facility	1,291 (91.6%)	42 (80.8%)	3.3	
Other	5 (0.4%)	1 (1.9%)	25.0	
All	1409 (100%)	52 (100%)	3.8	
Number of ANC visits				
None	5 (0.4%)	0 (0.0%)	0	P=0.151
1–2	125 (8.9%)	5 (9.6%)	4.1	
3	432 (30.8%)	23 (44.2%)	5.4	
4	634 (45.3%)	21 (40.4%)	3.4	
5 +	205 (14.6%)	3 (5.8%)	1.5	
All	1409 (100%)	52 (100%)	3.8	
Exclusive breast feeding				
Yes	733 (52.0%)	27 (51.9%)	3.7	P=1.000
No	676 (48.9%)	25 (48.1%)	3.8	
All	1409 (100%)	52 (100%)	3.8	

*Fisher exact test

Estimating the Adjusted Odds Ratio (95% Confidence Intervals) for HIV exposure and primary caregiver characteristics, we found that the lowest exposure rates were associated with mothers who were married, had education levels higher than primary school and were currently enrolled in college. Higher exposure rates were found for mothers older than 25 years of age and those who were widowed. The lowest rate of positive PCR test results were associated with the mother being older than 25 years of age, delivery using health facilities and the mother having higher than primary school level education (Table 5).

**Table 5:** Estimated adjusted Odds Ratio and 95% Confidence Intervals for infant HIV exposure MTCT

PMTCT interventions/characteristic of primary caregiver	HEI	HII
Sex of infant		
Male		1
Female		0.4 (− 0.2,1.0 )
Age of mother		
Less than 25	1	1
25–29	0.7*** (0.5,0.8)	− 1.1** (− 1.9,− 0.3)
30 +	1.2*** (1.0,1.3)	− 1.2*** (− 1.9,− 0.5)
Current vaccination		
Penta 1		1
Penta 2		− 0.5 (− 1.5,0.4)
Penta 3		− 0.7 (− 1.7,0.3)
Measles		0.6 (− 0.6,1.9)
Place of delivery		
Home		1
Health facility		− 0.9* (− 1.8,− 0.1 )
Other		1.4 (− 1.0,3.8)
Number of ANC Visits^a^		
1–2		1
3		0.7 (− 0.4,1.8)
4		0.1 (− 1.1,1.2)
5 +		− 0.9 (− 2.3,0.6)
Exclusive breast feeding		
Yes		1
No		− 0.5 (− 1.5,0.6)
Marital status*		
Single	1	1
Cohabiting	0.4 (− 0.2,1.1)	1.1(− 0.6,2.7)
Married	− 0.2**(− 0.4,− 0.1)	− 0.3(− 1.1,0.4)
Divorced	0.3(− 0.5,1.0)	
Separated	0.4(− 0.2,1.0)	1.3(− 0.3,2.9)
Widowed	1.3**(0.5,2.1)	0.7(− 1.4,2.8)
Highest level of Education		
Primary	1	1
Secondary	− 0.2** (− 0.3,− 0.1)	− 0.7*(− 1.3,− 0.0)
Higher	− 0.2 (− 0.5,0.0)	0.8 (− 0.2,1.9)
None	− 0.2 (− 0.5,0.1)	-1.1(− 3.1,0.9 )
Occupation (employment status)^a^		
Not employed	1	1
Formal employment	0.0(− 0.2,0.3)	− 1.3(− 2.7,0.1)
Self-employed/business	0.1(− 0.0,0.2)	− 0.1 (− 0.8,0.6)
In school	− 1.6* (− 3.1,− 0.2)	
Other	0.7 (− 0.0,1.5)	-0.1(− 2.5,2.3 )

^a^Excluded from the HII analysis were those who did not go for ANC (5), were divorced (9) or in school(2). N for HEI (8042), HII (1361). *p<0.05,**p<0.01, ***p<0.001

## Discussion

The study tested the feasibility of infant testing in immunization settings as a technique to determine MTCT rates among infants. The findings indicate that it is feasible, provided adequate resources for supplies (HIV test kids and reagents) are made available to cater for massive tests of all infants in immunization clinics. This will take a strain on existing supplies and will need adequate planning to ensure that all infants in immunization clinics are tested for exposure and all exposed infants are tested for HIV using DNA PCR. The success of the program, as indicated by previous studies will also depend on a strong data capture and documentation system, and robust routine data quality assessment (RDQA) [8, 10, 17–19].

Our study is the first to test the feasibility of this approach to determine MTCT rates in Zambia. However, this method is recommended by the World Health Organisation [20] and has been used and proven to be feasible in Malawi [21] and KwaZulu Natal in South Africa [22]. Our study makes a significant contribution to this growing body of knowledge and demonstrates the feasibility of using this approach to estimate HIV MTCT rates by other countries in the region. The study recruited an even distribution of babies across the four immunization types (Penta 1, Penta 2, Penta 3 and Measles) below the age of one year.

The transmission rate at 6 weeks stood at 4.7% and was consistent with the 2017 Joint United Nations Programme on HIV/AIDS (UNAIDS) estimate of 5%. The MTCT rate between 6 weeks and 9 months stood at about 3.8%, lower than the reported rates in earlier studies. Copperbelt (3.9%) and Lusaka (4.2) provinces had the highest overall proportion of HIV exposed babies who tested positive to HIV. This is in line with the national HIV prevalence trends which are high in Lusaka (15.7%) and Copperbelt provinces (13.8%) [14].

As expected, the highest rate of vertical transmission was detected among infants who came for Measles vaccination (5.0%) This was followed by those who came for Penta 1 (4.7%). It is likely that babies continue to become infected at all stages of immunization. This implies that the risk of infection is continuous and interventions are required throughout the PMTCT cascade until breastfeeding cessation. The majority of infants who tested PCR positive are of mothers below the age of 25 years. This is suggestive of the treatment naive mothers who either recently became aware of their status or are struggling to settle in their new sero-status.

In the multivariate analysis, we found a higher rate of infant exposure for mothers (1) older than 25 years of age, (2) unmarried and (3) with primary school level education. The association was significant for older mothers, those who were widowed (when compared to those who were single mothers), those who had secondary school education and those enrolled in college (compared to those who were unemployed). A higher rate of MTCT was associated with (1) female infants, (2) mother being younger than 25 years of age, (3) home delivery, (4)lower than 5 ANC visits, (5) exclusive breastfeeding, (6) primary education schooling attainment and (7) unemployment (Table 5). The MTCT association was significant for maternal age, place of delivery and secondary school attainment of the mother.

The study was successful in estimating the MTCT rates for infants below the age of 12 months thus indicating that infant testing in immunization services is a feasible strategy for estimating MTCT rates among the population of HIV exposed infants. Success of this strategy however requires mobilisation of resources and focusing them on inter alia; timely procurement and delivery of infant testing supplies, training on infant testing, timely testing of DBS and communication of results, establishment of robust RDQA to ensure that infant testing data is available on time, complete, accurate, reliable and is of high precision and integrity [23], and improving follow of mother–baby pairs [24].

The findings of this study must be interpreted within the following three limitations: The first limitation of this approach is that it misses some infants who may never be brought to the clinic for their immunizations during the period of data collection for different reasons. In addition, infants brought by caregivers other than their biological parents were excluded from the study. Although these constituted less than 1%, it biases the sample of infants included in the study. Therefore, not including HIV exposed children who never made it to the clinic for immunization or excluded for different reasons may underestimate the true MTCT rate. This limitation was also reported elsewhere [1].

The second limitation of this study relates to its cross-sectional nature. Because the study did not have a follow-up component for surveyed mother–baby pairs, it is not possible to distinguish early transmission from post-natal transmission. This distinction is important for Zambia because it is a breastfeeding nation [20].

The third limitation is that HIV status and ART uptake data were not collected from mothers due to the unreliability of such self-reported data. This limits the number of variables for multivariate analysis to isolate which factors contribute to infant HIV exposure and infection [20].

## Conclusion

This study suggests that it feasible to use immunization clinics to monitor both the maternal and infant HIV infections rates and that routine testing in immunization clinics can be used to identify exposed and infected infants early and link them to treatment. This finding is consistent with studies that have been conducted in the region and thus can be used by other countries in the region with high immunization coverage to estimate MTCT rates. Follow-up mechanisms however, need to be put in place to ensure that mother–baby pairs who are not enrolled in the HIV treatment program are tracked and linked to treatment and care. There is further need for routine data quality assessment of PMTCT programme data in all facilities providing PMTCT services. Although this will require increased resources to implement, the long-term cost of estimating MTCT rates and evaluating the effectiveness of the PMTCT program will be reduced.

## Data Availability

The datasets used and/or analysed during the current study are available from the corresponding author on reasonable request.

## Abbreviations

ANC: Antenatal care
ART: Antiretroviral treatment
BCG: Bacille Calmette Guerin
DBS: Dried blood spot
DNA: Deoxyribonucleic acid
DPTHibHep: Diphtheria, pertussis, tetanus, haemophilus influenza type B, hepatitis B
HEI: HIV exposed infant
HII: HIV infected infant
HIV: Human immunodeficiency virus
IPV: inactive polio vaccine
MTCT: Mother-to-child transmission
OPV: Oral polio vaccine
PCR: Polymerase chain reaction
PCV: Pneumococcal conjugate vaccine
PMTCT: Prevention of mother-to-child transmission
RDQA: Routine Data Quality Assessment Joint United Nations Programme on HIV/AIDS (UNAIDS)
